# Synergistic effects of antimicrobial components of the human-derived composite amnion-chorion membrane on bacterial growth

**DOI:** 10.3389/fcimb.2024.1472737

**Published:** 2024-10-07

**Authors:** Alexandra Su Brummerhop, Chun-Teh Lee, Robin Weltman, Gena D. Tribble, Ransome van der Hoeven, Yulun Chiu, Jianming Hong, Bing-Yan Wang

**Affiliations:** ^1^ Department of Periodontics and Dental Hygiene, The University of Texas Health Science Center at Houston School of Dentistry, Houston, TX, United States; ^2^ Department of Clinical Sciences, University of Nevada School of Dental Medicine, Las Vegas, NV, United States; ^3^ Department of Diagnostic and Biomedical Sciences, The University of Texas Health Science Center at Houston School of Dentistry, Houston, TX, United States; ^4^ Iowa Institute for Oral Health Research, University of Iowa College of Dentistry, Iowa City, IA, United States; ^5^ Department of Periodontics, University of Iowa College of Dentistry, Iowa City, IA, United States; ^6^ Department of Melanoma Medical Oncology, The University of Texas MD Anderson Cancer Center, Houston, TX, United States

**Keywords:** amnion, antimicrobial peptides, chorion, guided tissue regeneration (GTR), periodontal

## Abstract

**Introduction:**

The human-derived amnion-chorion membrane (ACM) has endogenous antimicrobial properties, which are important for preventing the colonization and survival of oral bacteria on exposed membranes. This project aimed to decipher the underlying mechanism by identifying the components of ACM that confer antibacterial properties. In addition, the antimicrobial efficacy of these identified components on oral bacteria was assessed.

**Methods:**

Four antimicrobial proteins, histone H2A/H2B, cathelicidin LL-37, lactoferrin, and lysozyme, were identified via mass spectrometry in ACM. These proteins were then assessed for their efficacy in killing *Streptococcus gordonii* Challis. Log-phased bacterial cells were cultured with the commercially available proteins that were identified in ACM, either individually or in combination, at different concentrations. After incubation for 8 or 24 hours, the bacteria were stained with a live/dead viability kit and analyzed via confocal microscopy.

**Results:**

The combination of these proteins effectively killed *S. gordonii* in a dose-dependent fashion after 8 or 24 hours of incubation. When each protein was tested individually, it killed *S. gordonii* at a much lower efficacy relative to the combinations. The synergistic effects of the antimicrobial protein combinations were also observed in both the viable cell count recovery and minimum inhibitory concentration assays.

**Discussion:**

By shedding light on the mechanisms in the ACM’s antimicrobial property, this study may raise more awareness of the potential benefit of utilization of a membrane with endogenous antimicrobial properties in regeneration surgeries.

## Introduction

There are different classes of naturally occurring antimicrobial polypeptides in the human body (Harder et al., 2007). Some of these proteins such as β-defensins serve as first line defenses against microbial infections. These natural antimicrobials are part of the innate immune system and are present in multiple tissues in the human body including tissues in the reproductive system. During pregnancy, the amniotic and chorionic membranes, besides their immunomodulatory properties, produce multiple antimicrobial components that protect the fetus from infections, including β-defensins, elafin, cathelicidin LL-37, secretory leukocyte protease inhibitor, histone proteins, and lactoferrin. This has been documented in many studies (Kanyshkova et al., 2001; Kjaergaard et al., 2001; Kim et al., 2002; Williams et al., 2006; King et al., 2007; Stock et al., 2007; Niknejad et al., 2008; Frew and Stock, 2011; Boldenow et al., 2016; Hoeksema et al., 2016; Mao et al., 2017; Tehrani et al., 2017; Zare-Bidaki et al., 2017).

The human-derived amnion-chorion membrane (ACM), which contains multiple growth factors, can promote tissue regeneration (Lei et al., 2017). Additionally, the documented antimicrobial properties of ACM can potentially prevent the adverse impact of contamination on regenerative outcomes and assist in reducing the use of antibiotics for surgeries (Holtzclaw and Tofe, 2017; Palanker et al., 2019). Recently, ACM has been utilized in periodontal tissue regeneration (King et al., 2007; Stock et al., 2007; Gupta et al., 2015). The antimicrobial properties of the ACM have been demonstrated in *in vitro* studies showing bacterial growth inhibition on the ACM (Ashraf et al., 2019; Palanker et al., 2019). Since BioXclude is a dehydrated human allograft that consists of laminated human amnion-chorion membranes that entered commercial production within the past two decades, the current study was carried out to determine the antimicrobial components in it, relative to those previously reported in non-processed ACM.

Four antimicrobial peptides/proteins, histone H2A/H2B, cathelicidin LL-37, lactoferrin, and lysozyme, were identified via mass spectrometry in ACM in this study. Histones are basic proteins in the nucleus of eukaryotic cell. They can also be found in the cytoplasm, cell membranes, extracellular fluid and function as antimicrobial peptides (Kim et al., 2002; Wang et al., 2011; Li et al., 2022). Cathelicidins are cationic antimicrobial peptides, produced by neutrophils, monocytes, dendritic cells and a subset of microphages (Mendez-Samperio, 2010; Koppen et al., 2019). Lactoferrin is an iron-binding glycoprotein of the transferrin family found in milk and other exocrine secretions, including saliva (Abd El Hafez et al., 2013; Woodman et al., 2018; Zarzosa-Moreno et al., 2020). Lysozyme is an enzyme that has ability to hydrolyze peptidoglycans in bacterial cell wall (Rouchon et al., 2022). All these four peptides/proteins are found in the oral cavity, especially in saliva (Amerongen and Veerman, 2002; Gorr, 2009; van 't Hof et al., 2014; Dawes et al., 2015; Salvatori et al., 2016; Khurshid et al., 2017). These natural antimicrobial proteins are the essential part of the innate immunity in the oral cavity that protect human body against bacterial infection.

Periodontal regenerative therapy allows restoration of the original architecture and function of the damaged periodontal tissue, as opposed to repair. Guided tissue regeneration (GTR) and guided bone regeneration (GBR) are commonly performed surgical procedures to regenerate tissues around teeth or bone ridges, respectively. These treatments involve the use of a barrier membrane to exclude the rapidly proliferating epithelial and connective tissue cells (Greenstein and Caton, 1993) and to create space to selectively promote the proliferation of progenitor cells to restore periodontal tissues (Nyman et al., 1987; Karring et al., 1993). The membrane also stabilizes the wound and augments the concentration of regenerative growth factors by sealing away the tissue space that is targeted for reconstruction (Linde et al., 1993). One of the most common complications of GTR and GBR is membrane exposure. An exposed membrane with oral bacterial adherence can jeopardize the healing process and compromise the clinical outcomes of tissue reconstruction (Machtei, 2001; Hung et al., 2002; Garcia et al., 2018; Lim et al., 2018). Due to the risk of membrane exposure and the negative impact of subsequent microbial contamination on regenerative outcomes, it would be beneficial to utilize a barrier membrane with antimicrobial properties.

The aim of this study was to identify the specific antimicrobial components in ACM and to determine whether the antimicrobial efficacy of ACM is mediated through the respective or synergistic activity of these components. The antimicrobial properties of ACM can be useful in the initial phase of wound healing, in which bacterial contamination and colonization impair the treatment outcome.

## Materials and methods

### Amnion–chorion membranes

The ACM used in all experiments was a commercial product (BioXclude^®^, Snoasis Medical, LLC, CO, USA) (Holtzclaw and Tofe, 2017). No human tissue samples were utilized in any of our experiments. The study has been approved by the UTHealth Institutional Biosafety Committee (IBC-22-066). The ACM used in all experiments is a commercial product. The amnion and chorion membranes are harvested from the placenta under sterile conditions with the donor’s consent, in accordance with the provisions of the Food and Drug Administration and the American Association of Tissue Banks. Harvested placental tissues are preserved while serological safety is confirmed. Following serological clearance, the amnion and chorion membranes are isolated from the placenta and processed via the proprietary Purion® process. The membranes are initially cleansed gently, followed by de-epithelialization of the amnion membrane. The de-epithelialized amnion membrane is then laminated onto the directly isolated chorion membrane. De-epithelialization enhances amnion-chorion membrane adhesion by exposing the basement membrane. Subsequently, the thicker (about 300μm) composite membrane undergoes chemical decontamination and dehydration under regulated conditions. This is followed by a terminal sterilization process utilizing gamma radiation and product packaging.

### Culture of the bacterial strain

*Streptococcus gordonii* Challis, a gram-positive bacterium and one of the initial colonizers in the oral cavity, was used as a representative early colonizer (Socransky et al., 1998; Huang et al., 2011) in this study. *S. gordonii* was inoculated onto Todd Hewitt agar plates (comprised of 3 g of dehydrated Todd Hewitt Broth (THB) and 1.5 g of agar (Difco, Detroit, MI, USA) in 100 ml of H_2_O) and cultured for 36 to 48 hours at 37°C in an anaerobic jar containing a bag of BD BBL CO_2_ generator (Becton, Dickinson and Company, Sparks, MD, USA) to create a CO_2_-enriched environment. The medium used for bacterial culture and experimental assays was THB.

### Bacterial preparation

*S. gordonii* colonies were transferred from THB agar plates to 1 ml of THB in an Eppendorf tube and grown overnight at 37°C to the stationary phase. *S. gordonii* overnight cultures were diluted in fresh 37°C prewarmed THB (1:4 dilution) and incubated for another hour at 37°C to log phase. The log phase *S. gordonii* was further diluted 100× in THB for the experimental assays.

### High-performance liquid chromatography and mass spectrometry

HPLC is a type of chromatography, a laboratory technique for the separation of a mixture. MS is an analytical technique that identifies compounds in samples by measuring the mass/charge ratio of their constituent molecules. Pieces of samples (1 mm × 1 mm) from three ACMs were digested in the solvent, 200 ng of modified trypsin (Promega, Madison, WI, USA) and Rapigest (Waters Corporation, Milford, MA, USA), for 18 hours at 37°C. The resulting peptides were analyzed by HPLC-MS/MS coupled with an Orbitrap Fusion mass spectrometer (Thermo Scientific^TM^, Waltham, MA, USA). Proteins and peptides in ACMs were identified by Mascot (v2.6, Matrix Science, London, UK) and Proteome Discoverer (v2.2, Thermo Scientific, San Francisco, US) and quantified with total ion count through Scaffold (v4, Proteome Software, Portland, US). The normalization algorithm applied in Scaffold normalizes spectral counting data of all data loaded in the experiment. The searches were performed with a precursor mass tolerance of 10 ppm and fragment ion mass tolerance of 0.8 Dalton using monoisotopic parent and fragment ion masses, allowing for up to two missed cleavages with trypsin digestion. The variable modifications were methionine sulfoxide and pyro-glutamate formation. The proteins and peptides were determined with a false discovery rate (FDR) <1.0% and were categorized by gene ontology (GO) terms.

### Glass-bottom well assay

Four commercially available proteins that were identified in the ACM analytes as described above were utilized in this assay to assess their antimicrobial efficacies, either individually or as mixtures of all 4 proteins. Each protein stock solution, lysozyme (100 mg/ml, Sigma Chemical Co., St. Louis, MO, USA), cathelicidin LL-37 (10 mg/ml, AnaSpec Inc., Fremont, CA, USA), lactoferrin (50 mg/ml, Athens Research & Technology Inc., Athens, GA, USA), or histone H2A/H2B dimer (20 µM, New England BioLabs, Ipswich, MA, USA), was mixed with log phase bacteria in Eppendorf tubes. The final concentrations in the individual-protein cultures were 10 mg/ml for lysozyme, 1 mg/ml for cathelicidin LL-37, 5 mg/ml for lactoferrin, and 2 µM for the histone H2A/H2B dimer. Each protein and 2 × 10^5^ *S. gordonii* cells in 55μl volume, were added to the wells of a glass-bottomed 96-well microtiter plate (Eppendorf North America, Hauppauge, NY, USA). For the synergistic assessment, a combination of lysozyme, cathelicidin LL-37, lactoferrin, and histone H2A/H2B dimer (in equal volumes) was mixed with the log phase bacteria at 1:10, 1:20, and 1:50 ratios. The mixtures were added to wells in glass-bottomed microtiter plates in volumes of 70 µl, 60 µl, and 54 µl, representing 100%, 50%, and 20% concentrations of each protein, respectively, relative to the individual protein experiments. The cell numbers per well were the same as those in the individual protein assays (2 × 10^5^ cells in each well). The negative controls were 2 × 10^5^ *S. gordonii* cells/well in THB without the addition of any protein. The positive controls were 6 mm circular pieces of ACM obtained using a biopsy punch (Miltex, Inc., York, PA, USA). The plates were incubated in a CO_2_-enriched environment for 8 or 24 hours at 37°C in a box with a water compartment. At each time point, 40 µl of 800X-diluted (in saline) Live/Dead BacLight Bacterial Viability Kit Component B (Thermo Scientific^TM^, Waltham, MA, USA) was added to each well and stained for 15 minutes at room temperature. All the experiments were performed in triplicate for each protein group, and the same experiments were repeated at least three times. The antimicrobial efficacy was determined using confocal microscopy and viable count recovery assays.

### Confocal microscopy imaging

Confocal analysis was performed using a Nikon C2plus confocal microscope and NIS Elements AR software (Nikon Instruments Inc., Melville, NY, USA) for visual data collection. The filter set and laser settings utilized were 488 nm (green) for FITC and 561 nm (red) for TRITC. One or two representative images of each well were captured at different magnifications (100X or 200X) after the entire area of the bottom layer was viewed.

### Viable count recovery assay

This assay was designed to quantify viable bacteria cultured with individual proteins or their combinations in varying dilutions. After confocal microscopy, the attached *S. gordonii* cells were scraped from the bottom of the glass well using a 1 ml pipet tip. The cells in the well were mixed well by pipetting and transferred to an Eppendorf tube. The bacteria were then sonicated five times at a setting of Output 3, Pulse, to break the bacterial chains (Sonic Dismembrator Model 100, Fisher Scientific, Hampton, NH, USA). Each sample was serially diluted in Hanks' balanced salt solution (HBSS, Thermo Scientific^TM^, Waltham, MA, USA), and 15 μl of each dilution was subsequently plated onto Mitis Salivarius Agar plates (Difco, Detroit, MI, USA). The plate was incubated at 37°C for 24 to 48 hours in an anaerobic jar in a CO2-enriched environment, and colony-forming units (CFU) were counted manually under a stereo microscope.

### Minimum inhibitory concentration

Concentrations of 20 mg/ml for lysozyme, 2 mg/ml for cathelicidin LL-37, 10 mg/ml for lactoferrin, and 4 µM for the histone H2A/H2B dimer were utilized to determine the MIC for each protein. For the combination of the proteins, the same concentrations as in the glass-bottom well assay (10 mg/ml for lysozyme, 1 mg/ml for LL-37, 5 mg/ml for lactoferrin, and 2 µM for the histone H2A/H2B dimer) were utilized to assess the MIC. The mixtures containing the proteins that were serially diluted in THB and 2 x 10^5^ *S. gordonii* cells in 60 µl and 70 µl in total volume for the single protein and the protein combination, respectively, were cultured at 37°C for 16 hours. The bacterial growth control (negative control) was 2 × 10^5^ *S. gordonii* cells in 60 µl and 70 µl of THB for the individual protein control and the protein combination control, respectively. Serially diluted tetracycline (10, 2, 0.4, 0.08, 0.016, and 0.0032 µg/ml) was utilized as the positive control. After incubation, the cultures were mixed by pipetting and transferred to cuvettes. The optical density of the samples was then determined using a spectrophotometer (Eppendorf, Biophtometer, Eppendorf North America, Hauppauge, NY, USA) at a wavelength of 600 nm. The MIC of each protein or their combination was defined as the lowest concentration that completely inhibited bacterial growth.

### Statistical analysis

For the viable bacterial cell count assessment, CFUs were compared among the control and protein groups using one-way analysis of variance (ANOVA) at 8-hour and 24-hour incubations, respectively. Comparisons between the means of individual proteins, combinations, and controls were analyzed by *post hoc* analysis (Tukey’s test). All values are expressed as the mean ± standard deviation. A p-value <0.05 was considered to indicate statistical significance.

## Results

### Identification of antimicrobial components in ACM

**Figure 1:**
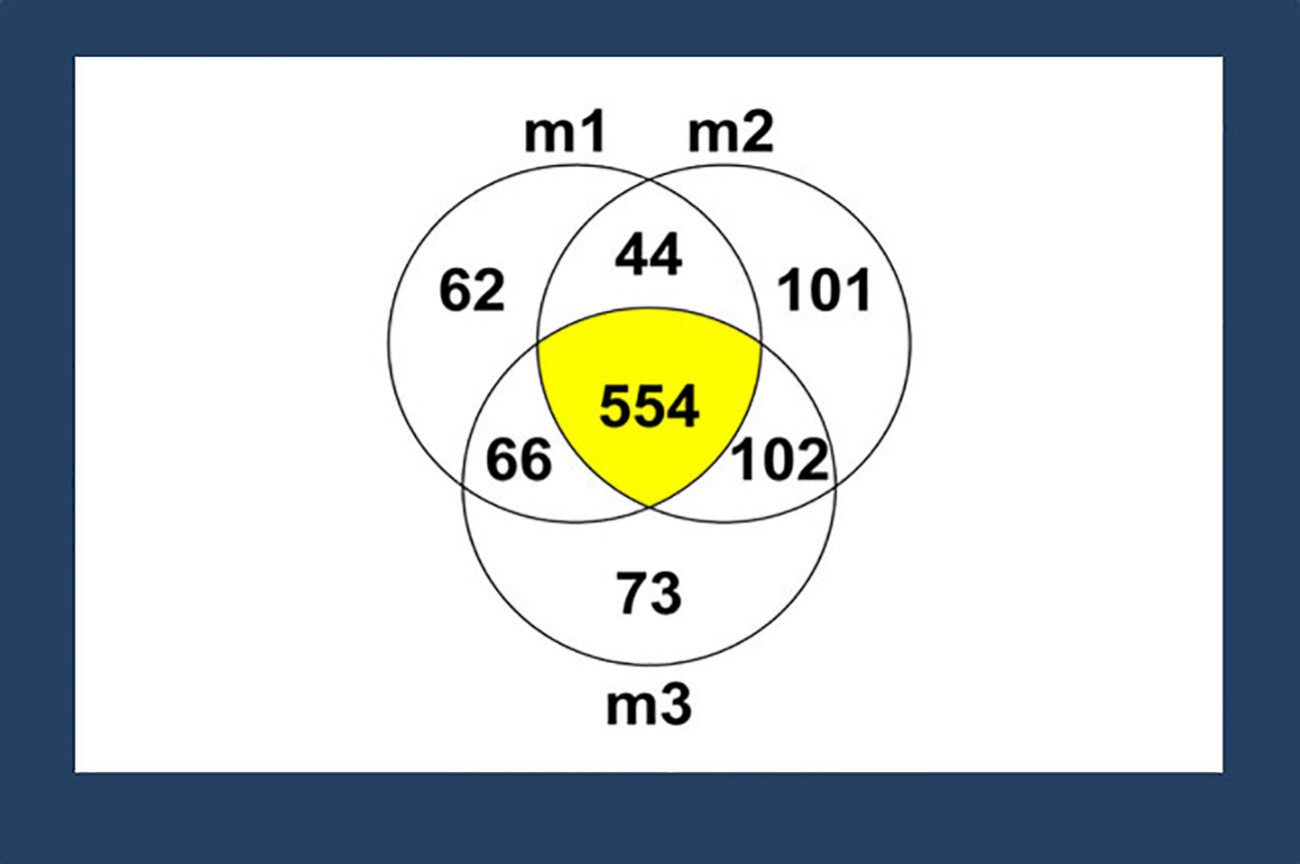
Proteins or peptides identified by mass spectrometry (MS). A total of 1007 proteins or peptides were identified in three different membranes tested (represented as m1, m2, and m3) via MS. Among those proteins or peptides, 554 proteins or peptides (55%) were shared among all the membranes. Proteins or peptides with antimicrobial properties -- lactoferrin, histone H2A, histone H2B, cathelicidin LL-37, and lysozyme, were identified based on a literature search.

HPLC-MS/MS identified 1007 proteins and peptides among the three ACMs, with over half of the proteins and peptides being shared by the three membranes. There is variability among the proteins and peptides in the membranes because the results are a semi-quantification where the presence of proteins or peptides is indicated by the isotopic composition of the elements in a molecule. The proteins with reported antimicrobial ability among these 1007 proteins and peptides are lactoferrin, histone H2A, histone H2B, cathelicidin LL-37, and lysozyme (**Figure 1**). Since these antibacterial molecules consist of more than 100 amino acids, they are mentioned as proteins here for consistency. However, some of these compounds have been described as peptides in the literature (Ramuta et al., 2021).

**Figure 2:**
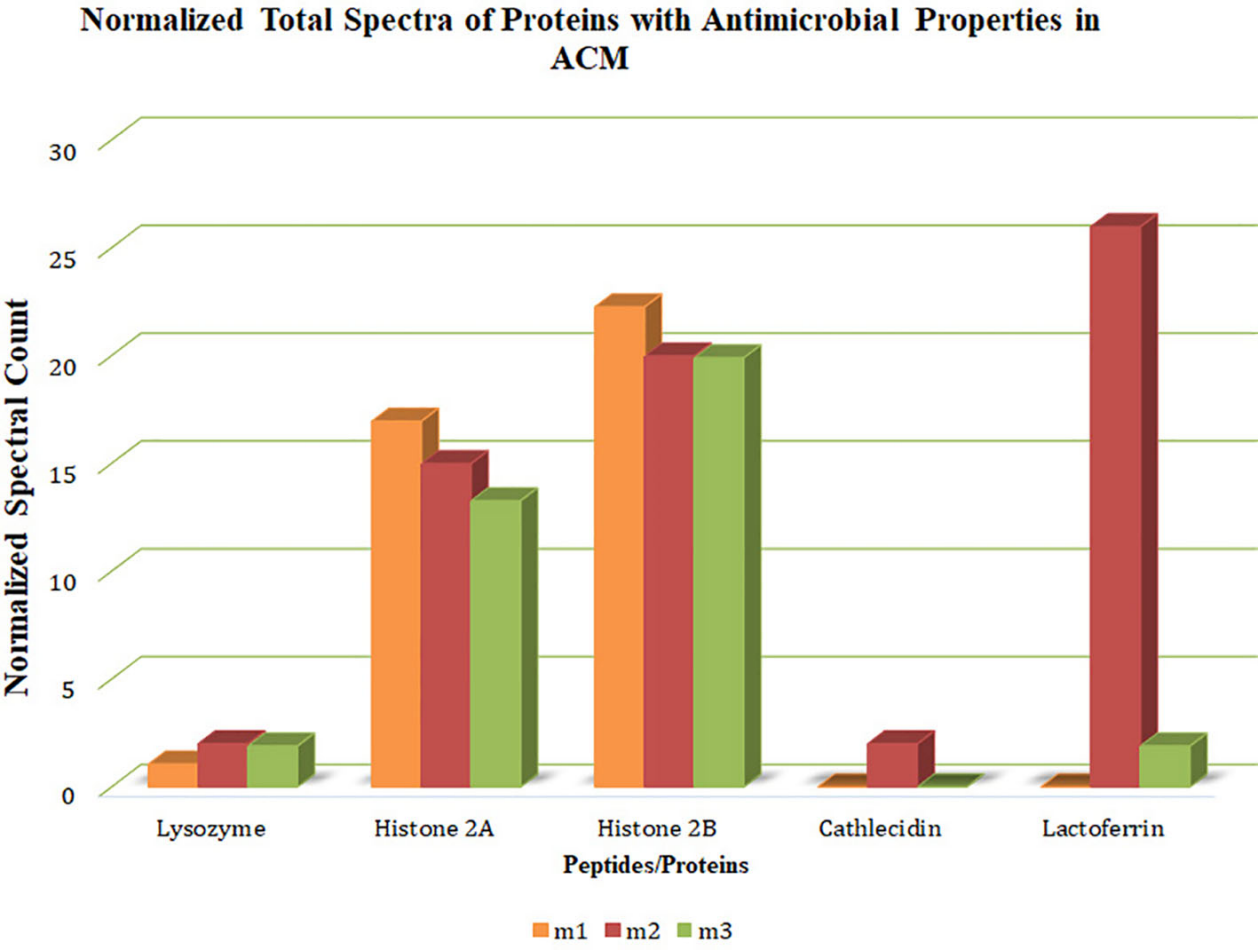
Quantitative values (normalized total spectra) of proteins. The quantitative value (normalized total spectra) of proteins with antimicrobial properties in the human-derived amnion-chorion membrane (ACM) is based on the fragments resulting from ionization and their signal intensity. M1/2/3: ACM sample 1/2/3.

The spectral counts of proteins or peptides with antimicrobial properties in ACM were determined based on the fragments resulting from ionization and their signal intensity (**Figure 2**). Histone H2A, histone H2B, and lysozyme were present in all three membranes. Cathelicidin LL-37 was present in one membrane, and lactoferrin was present in two membranes. Generally, histone H2A and histone H2B had a higher count and more homogenous presence than others.

### Demonstration of the antimicrobial ability of the proteins in killing *S. gordonii* in glass-bottom well assays

**Figure 3:**
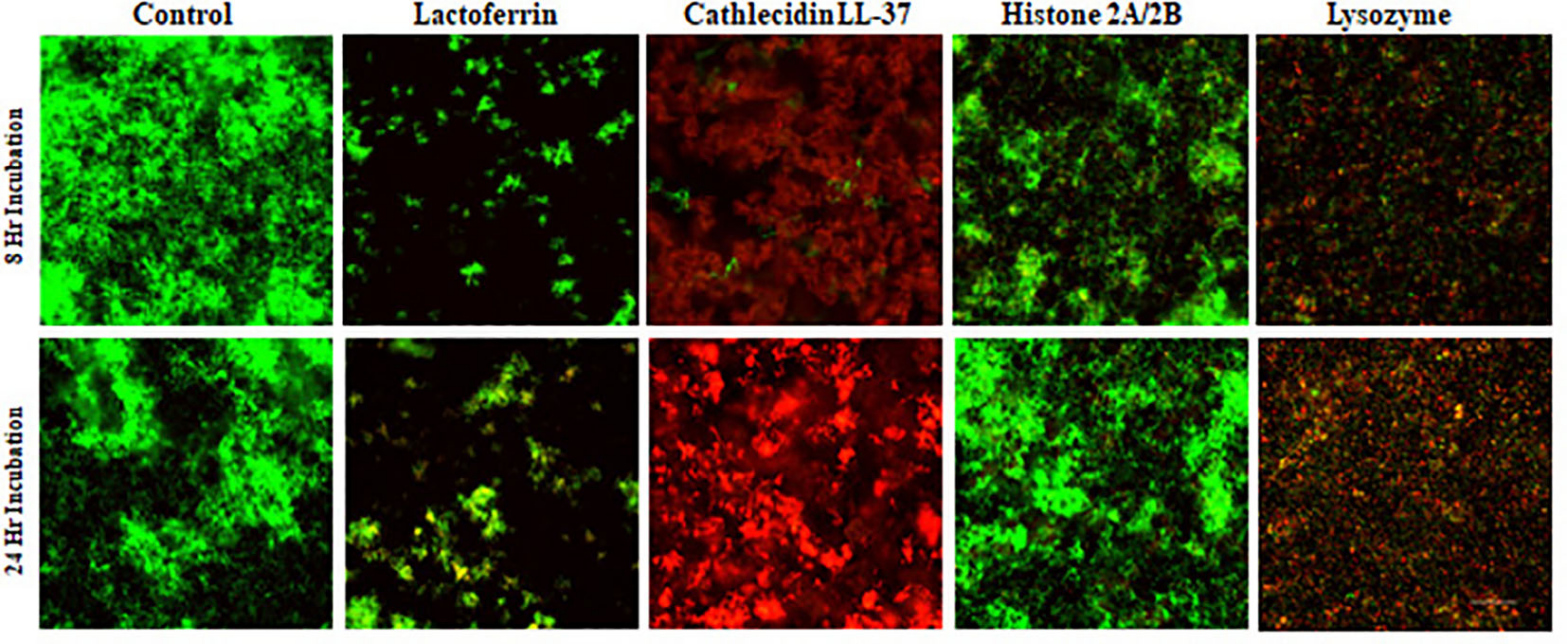
Confocal images of assessing antimicrobial ability of individual protein or peptide. *S. gordonii* cells were cultured with individual antimicrobial protein or peptide for 8 or 24 hours, Live/Dead stained, and analyzed with confocal microscopy at 200x magnification. Green represents live bacteria (SYTO9), and red indicates dead bacteria (propidium iodide). Scale bar: 100μm.

*S. gordonii* typically presents as long chains of cocci (**Figure 3**, Control). For individual proteins, lactoferrin treatment resulted in fewer bacterial cells relative to the control, with the majority of them alive (**Figure 3**, Lactoferrin), indicating that lactoferrin mainly inhibited bacterial growth at both time points. Cathelicidin LL-37, on the other hand, exhibited bactericidal ability, killing the majority of the cells (**Figure 3**, Cathelicidin LL-37) at 8 hours and even more at 24 hours. Lysozyme disrupted the long chains and killed most bacteria. In contrast to the other three proteins, *S. gordonii* thrived in the presence of the histone H2A/H2B dimer at 8 and 24 hours, indicating minimal antimicrobial activity against *S. gordonii*.

**Figure 4:**
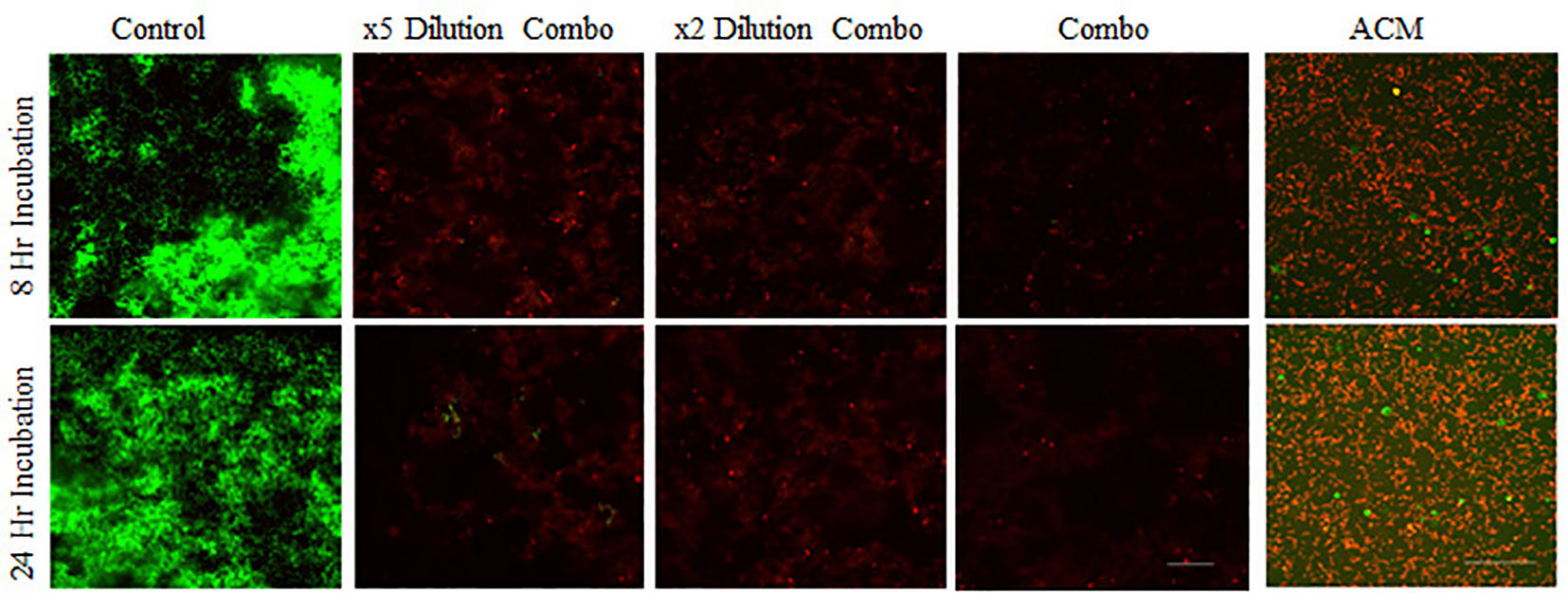
Confocal images of assessing the antimicrobial ability of protein and peptide combinations. *S. gordonii* cells were cultured with mixtures of lactoferrin, LL-37, histones, and lysozyme at the same concentration as in individual assays, at their x2 dilution, and at x5 dilutions, and with 6 mm diameter ACM for 8 or 24 hours, Live/Dead stained, and analyzed with confocal microscopy at 200x magnification. Green represents live bacteria (SYTO9), and red indicates dead bacteria (propidium iodide). Scale bar: 100μm.

The combinations of the four proteins, even at 1/5 of the concentrations used for the single protein, demonstrated significant bactericidal activity. Similar to the protein combinations, the intact ACM also resulted in the complete absence of live bacteria (**Figure 4**). The large round green dots in **Figure 4** of the ACM were from the staining solution, based on the shapes and large diameters. The results of glass-bottom well assays were further confirmed with bacterial viability cell count assays (see below).

### Bacterial viability cell count assays

To confirm the confocal analysis results and quantify the living *S. gordonii* cells, the bacterial viability was determined. Lactoferrin, cathelicidin LL-37, and lysozyme at the indicated concentrations significantly reduced the survival of *S. gordonii* at both time points relative to that of the controls and histones, via one-way ANOVA analysis or the subsequent *post hoc* test (p <0.001) (**Table 1**).

**Table 1:** Bacterial viability cell count for assessing antimicrobial ability.

	8 Hour Incubation	24 Hour Incubation
**Control (THB)**	2.54 x 10^7^ ± 1.14 x 10^6^	3.95 x 10^7^ ± 4.67 x 10^5^
**Histone H2A/H2B (2 µM)**	1.52 x 10^7^ ± 4.67 x 10^5^	2.74 x 10^7^ ± 5.26 x 10^6^
**Lactoferrin (5 mg/ml)**	2.77 x 10^5^ ± 3.06 x 10^4^	2.66 x 10^5^ ± 1.24 x 10^4^
**LL-37 (1 mg/ml)**	2.48 x 10^5^ ± 1.20 x 10^5^	5.50 x 10^2^ ± 2.59 x 10^2^
**Lysozyme (10 mg/ml)**	2.81 x 10^5^ ± 1.09 x 10^4^	8.95 x 10^4^ ± 5.50 x 10^4^
**ACM**	0 ± 0	0 ± 0
**Combo**	0 ± 0	0 ± 0
**2X Dilution**	0 ± 0	0 ± 0
**5X Dilution**	2.86 x 10^3^ ± 2.55 x 10^1^	4.41 x 10^4^ ± 1.50 x 10^4^

The viable cell count was carried out as a parallel assessment immediately after confocal imaging. Compared with those of the controls and histones, lactoferrin, cathelicidin LL-37, and lysozyme at the indicated concentrations significantly reduced *S. gordonii* at both time points (p <0.0001). The protein combination, 2X dilution of the protein combination, and ACM completely eliminated live *S. gordonii* in the cultures at both incubation time points. Compared with the controls, the 5X dilution of the protein combination significantly reduced *S. gordonii* at both time points (p< 0.0001). All values are expressed as the mean ± standard deviation. These p values were generated from one-way ANOVA and subsequent post hoc analysis with Tukey’s test.

The enhanced bactericidal effect of the protein combination was evident (**Table 1**). The protein combination (at the same concentrations as those of the individual proteins) and its 2X dilution completely eliminated *S. gordonii* in the cultures at both incubation time points. Even its 5X dilution killed comparable amounts of individual lactoferrin, cathelicidin LL-37, and lysozyme (**Table 1**). In addition, ACM also resulted in 0% survival of *S. gordonii* in the cultures at both incubation time points (**Table 1**).

### Determination of the minimum inhibitory concentration

The MIC test establishes the lowest concentration of an antimicrobial agent that prevents the visible growth of a microorganism. The MIC values for each protein group are shown in **Table 2**. Histone H2A/H2B dimers, even at a concentration of 4 µM, were not able to inhibit *S. gordonii* growth and, therefore, did not reach the MIC. This finding is in agreement with the results of the confocal analysis and viable cell counts. *S. gordonii* formed precipitates immediately upon mixing with cathelicidin LL-37, even at the lowest concentration (0.06 mg/ml) after serial 2X dilutions. This prevented us from obtaining a reliable OD value. The MICs for lactoferrin and lysozyme were 10 mg/ml and 20 mg/ml, respectively. For the protein combination group, the presence of cathelicidin LL-37 in combination did not cause any precipitation of the *S. gordonii* cells, even at the highest concentrations. A much lower concentration of each protein was required for the combination MIC relative to the individual protein assays (**Table 2**).

**Table 2:** Determination of antimicrobial protein minimum inhibitory concentration (MIC).

Proteins	MIC
**Histone H2A/H2B dimer**	N/A
**Lactoferrin**	10 mg/ml
**Lysozyme**	20 mg/ml
**Cathelicidin LL-37**	N/A
**Lactoferrin + Lysozyme + Cathelicidin LL-37**	5 mg/ml + 10 mg/ml + 1 mg/ml
**Lactoferrin + Lysozyme + Cathelicidin LL-37 + Histone H2A/H2B dimer**	1.8 mg/ml + 3.5 mg/ml + 0.35 mg/ml + 0.7 µM

N/A, not available

The MICs against *S. gordonii* for each protein or combination of proteins were determined by a spectrophotometer at a wavelength of 600 nm. These results indicate that the lowest concentrations of the proteins that completely inhibited *S. gordonii* growth occurred after 16 hours of incubation.

Since the histone H2A/H2B dimer did not exhibit obvious antimicrobial activity against *S. gordonii* in any of the assays (confocal analysis, viable cell count, and MIC test), the MIC of the three-protein combination without the presence of histones was further assessed. The MIC for the three-protein combination was approximately three times greater than that for the four-protein combination (**Table 2**), indicating the synergistic effect of the histone H2A/H2B dimer in the protein combination in eliminating *S. gordonii.*

## Discussion

This study aimed to investigate the mechanisms underlying the antibacterial properties of ACM. The results demonstrated that the ACM has antibacterial proteins and that these proteins could effectively inhibit bacterial growth or kill bacteria *in vitro*. The combination of these proteins even had better antimicrobial efficacy than the individual proteins.

The antibacterial characteristic of ACM is one of its valuable biological properties desirable in various medical and dental applications. Many antimicrobial peptides and proteins, including β-defensins-1, -2, and -3, elafin, cathelicidin LL-37, secretory leukocyte protease inhibitor (SLPI), and histone H2A and histone H2B, have been identified in unprocessed amnion and chorion membranes (Kanyshkova et al., 2001; Stock et al., 2007; Boldenow et al., 2013; Hoeksema et al., 2016; Ramuta et al., 2021). Studies have also demonstrated the antimicrobial properties of these membranes against various bacteria, including *Eschershelli coli, Bacillus cereus, Klebsiella pneumoniae, Enterococcus faecalis, Streptococcus pyogenes, Pseudomonas aeruginosa, Staphylococcus* spp.*, Acinetobacter calcoaceticus, Shigella flexneri*, and *Lactobacillus plantarum* (Kjaergaard et al., 2001; Tehrani et al., 2013; Zare-Bidaki et al., 2017). A previous study also showed that the specific processed ACM used in this study exhibited high efficacy against *S. gordonii* (Palanker et al., 2019). However, its antimicrobial mechanism is unclear.

This study utilized several assays to investigate the impact of proteins identified in the ACM on oral bacterial growth. Confocal microscopy is an imaging technique that enables the detailed visualization of cells through image optimization (Peterson, 2014). The confocal analysis clearly visualized the dead and live bacterial cells *in situ* immediately after incubation. However, *S. gordonii* could not be quantified because multiple *S. gordonii* coccal cells form long chains in cultures. Unless all the coccal cells were alive or dead, it would be extremely difficult to accurately determine the percentage of live or dead cells in each population. Therefore, the viable cell recovery assay was conducted as a parallel assessment to provide more information on the bactericidal activity of the antimicrobial proteins. In this study, confocal imaging was used for qualitative analysis, and quantitative analysis was performed via a viable cell recovery assay. Both assays showed the corresponding antibacterial effects of the tested proteins.

Lactoferrin, a glycoprotein, exhibited predominant bacteriostatic activity with limited bactericidal activity on the oral bacteria tested. Lactoferrin has been shown to exert its bacteriostatic effect through an iron-dependent mechanism involving the sequestration of iron ions (Kim et al., 2002). Lactoferrin can also kill bacteria through binding to lactoferrin receptors, such as the Lipid A moiety of lipopolysaccharides (LPS), on the surface of microorganisms, resulting in the release of LPS from microbial cell walls (Kanyshkova et al., 2001; Orsi, 2004). Our results demonstrated that lactoferrin effectively inhibited the growth of *S. gordonii*, an early colonizer in the oral cavity. The histone H2A/H2B dimer exhibited minimal evidence of cell death after 8 and 24 hours of incubation. Histone H2A and histone H2B are involved in the formation of neutrophil extracellular traps (NETs), a scaffold consisting of different kinds of proteins and DNA in which bacteria are trapped and killed (Kim et al., 2002; Hoeksema et al., 2016). Histone H2B can also induce microbial death by binding to bacterial DNA (Kim et al., 2002) or neutralizing bacterial endotoxins (Hoeksema et al., 2016), suggesting that a longer incubation period may be required before its antibacterial activity can take effect. On the other hand, cathelicidin LL-37 demonstrated significant bactericidal activity against the bacteria tested. Cathelicidin LL-37 exerts its bactericidal effect by disrupting the bacterial membrane of gram-negative and gram-positive bacteria through the formation of pores (Duplantier and van Hoek, 2013; Xhindoli et al., 2016), which may explain the killing effect in this study. Finally, lysozyme demonstrated bactericidal effects that were more predominant in the late incubation phase. Lysozyme hydrolyzes peptidoglycan on the bacterial cell wall via its neuraminidase moiety, causing cell wall degradation and bacterial lysis (Yadav et al., 2017). Compared with gram-negative bacteria, it has a greater antibacterial effect on gram-positive bacteria due to the presence of a significantly greater number of peptidoglycan layers on their cell walls (Masschalck and Michiels, 2003).

The combined proteins showed remarkable evidence of bactericidal activity relative to the individual proteins, with no evidence of viable *S. gordonii* after both 8 and 24 hours. This suggests that the combined antibacterial activity of the biomolecules was significantly greater than that of the respective individual molecules. Therefore, antimicrobial proteins appear to have synergistic antibacterial effects on *S. gordonii.* This synergistic effect was also shown by the complete elimination of *S. gordonii* by the intact ACM that has various antimicrobial proteins.

In this study, a spectrophotometric technique using a cuvette instead of a microplate was used to determine the MIC due to the small total volume of each sample. This technique is based on the principle of Beers’ law, which states that the absorbance of a medium is directly proportional to the quantity of the substance (bacteria) absorbing the light (Akinduti et al., 2019). Although the histone H2A/H2B dimer did not inhibit bacterial growth as an individual protein in the MIC assay, it did enhance the antimicrobial efficacy of protein combinations. This was demonstrated by the lower MIC of the all-protein combinations relative to that of the three-protein combinations, which indicates a contributory role of histone H2A and histone H2B in the antibacterial property of ACM. In general, the protein combinations exhibited a lower MIC than each individual protein, which is in accordance with the results of the confocal microscopy and viable cell recovery count assays. These findings suggest a synergistic antibacterial mechanism.

The initial concentrations used to determine the MICs of the individual proteins (20 mg/ml for lysozyme, 2 mg/ml for cathelicidin LL-37, 10 mg/ml for lactoferrin, and 4 µM for the histone H2A/H2B dimer) were two times greater than those utilized in the confocal and bacterial viability assays. These starting concentrations were chosen since the lower concentrations of these proteins (lysozyme, cathelicidin LL-37, and lactoferrin) used in confocal and bacterial viability assays significantly killed *S. gordonii*. Although 4 µM histone H2A/H2B dimers did not inhibit bacterial growth, a higher concentration was not used in the MIC assay because it was already 20% of the final volume of the cultures.

BioXclude membrane is a dehydrated human allograft that consists of laminated human amnion-chorion membranes. As described in Materials and Methods, de-epithelialization of the amnion membrane was de-epithelialized during the production of the commercially available membrane. The de-epithelialized amnion membrane is then laminated onto the directly isolated chorion membrane. This de-epithelialization of the amnion membrane might result in the absence of defensins, which have been widely reported in previous studies of non-processed ACM.

This study had several limitations. The results of mass spectrometry showed the heterogeneity of proteins and peptides identified in the ACM, which could be associated with membranes processed from different donors. This heterogeneity may cause variations in biological attributes (Lei et al., 2017). Due to the limited number of tested ACMs, it is possible to overlook other antimicrobial proteins. The concentrations of proteins used in these experiments may not be the same as those present in the commercial ACMs. In the future, the amounts of these antimicrobial proteins in these commercial ACMs can be quantified using other methods, such as enzyme-linked immunosorbent assay (ELISA). Further research is needed to characterize the influence of human saliva on the antimicrobial effects of the ACM in an environment mimicking the oral cavity.

## Conclusion

The specific antimicrobial proteins, lactoferrin, lysozyme, cathelicidin LL-37, and the histone H2A/H2B dimer that can contribute to the antibacterial properties of ACM, were identified. While each of the proteins has respective antibacterial properties, the combination of these four proteins killed oral *S. gordonii* more efficiently than any single protein, indicating a synergistic mechanism in ACM. This study provided an explanation for the antimicrobial properties of ACM, which may be important for preventing poor treatment outcomes associated with membrane exposure and bacterial contamination.

## Data Availability

The original contributions presented in the study are included in the article/supplementary material. Further inquiries can be directed to the corresponding author.
